# Hemangiopericytoma following allogeneic hematopoietic stem cell transplantation in a patient with primary plasma cell leukemia: the first case report and literature review

**DOI:** 10.3389/fonc.2024.1467237

**Published:** 2024-11-14

**Authors:** Yajun Shi, Guohao Liang, Haiying Zhang, Yaqing Wang, Ying Han, Shenglian Tong, Shunyu Liang, Ying Wang, Hai Bai, Rui Xi

**Affiliations:** ^1^ Department of Hematology, The 940th Hospital of Joint Logistics Support Force of Chinese People’s Liberation Army, Lanzhou, China; ^2^ Department of Pathology, The 940th Hospital of Joint Logistics Support Force of Chinese People’s Liberation Army, Lanzhou, China; ^3^ Department of Diagnostic Radiology, The 940th Hospital of Joint Logistics Support Force of Chinese People’s Liberation Army, Lanzhou, China

**Keywords:** primary plasma cell leukemia, allogeneic hematopoietic cell transplantation, secondary tumors, hemangiopericytoma, case report

## Abstract

**Background:**

Hemangiopericytoma (HPC) is a rare secondary tumor after allogeneic hematopoietic stem cell transplantation (allo-HSCT), which has not been reported in the literature. Herein, we reported a case of HPC after allo-HSCT.

**Case description:**

We reported a case of a middle-aged female patient with primary plasma cell leukemia who presented lumbosacral pain and right lower limb pain and numbness on1684 days post-transplant. She underwent an MRI of the lumbar spine, which showed abnormal signal intensity in the spinal canal at the second through fifth lumbar spine vertebral levels. The patient was diagnosed with HPC based on a pathological biopsy of the diseased tissue in the spinal canal. Radiotherapy was administered to the lesion in the second through fifth lumbar vertebrae. The patient experienced less numbness and pain.

**Conclusion:**

According to the literature, this is the first reported case of post-transplant HPC. Therefore, attention should be paid to secondary tumors after transplantation, especially rare tumors.

## Introduction

Secondary tumors after allogeneic hematopoietic stem cell transplantation (allo-HSCT) have become an important factor affecting the long-term survival of patients (1). Patients after allo-HSCT have a 1.7-2.1 times higher risk of developing non-hematological second malignancies than the general population (2, 3). 5-10% of late deaths after allo-HSCT are caused by secondary tumors. Secondary tumors after allo-HSCT include cancers of the esophagus, thyroid and skin (2, 4, 5). However, to our knowledge, hemangiopericytoma (HPC) after hematopoietic stem cell transplantation (allo-HSCT) has not been reported. Herein, we report a first case of HPC after allo-HSCT, detailing the clinical and pathologic findings, treatment and follow-up. Additionally, the literature related to HPC was reviewed.

## Case description

### Clinical findings

**Figure 1:**
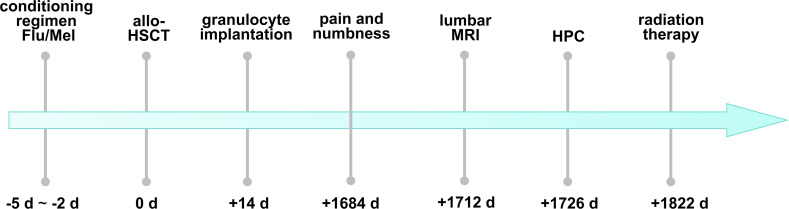
The timeline of the patient’s discomfort after allo-HSCT and the diagnosis and treatment of HPC. Flu, fludarabine; Mel, melphalan; allo-HSCT, allogeneic hematopoietic stem cell transplantation; MRI, magnetic resonance imaging; HPC, hemangiopericytoma.

A 48-year-old woman was diagnosed with primary plasma cell leukemia by bone marrow morphology and immunotyping (**Figure 1**). Her karyotype is 46, XX. The patient underwent bortezomib, doxorubicin liposomes, dexamethasone (PAD) chemotherapy regimen and achieved complete remission. The specific drugs and dosages of PAD regimen are bortezomib 2mg on d1, d4, d8, d11, doxorubicin liposomes 40mg on d1, dexamethasone 40mg on d1-4, d8-11. She received four 21-d cycles of PAD chemotherapy regimen. The patient went into complete remission as evidenced by a bone marrow biopsy. Then, she underwent HLA-matched sibling transplantation from her brother. The conditioning regimen consisted of fludarabine 50mg/d (-5d, -4d, -3d, -2d) and melphalan 150mg/d (-4d, -3d). She received 5.87×10^8^ mononuclear cells/kg and 2.34×10^6^ CD34^+^ cells/kg. The patient received a combination of cyclosporine, mycophenolate mofetil, and short-course MTX to prevent graft versus host disease (GVHD) after allo-HSCT. The patient underwent granulocyte implantation on day 14 and platelet implantation on day 16 after allo-HSCT. She underwent bone marrow cell morphology on day 28 after allo-HSCT and the result showed a complete remission. The patient’s chimerism was complete donor type. Since then, the patient’s condition has been stable at regular check-ups. She received lenalidomide maintenance therapy until one year after allo-HSCT. The patient developed oral chronic GVHD after 13 months of transplantation, which resolved after treatment with prednisone. Since then, the patient has led a normal life.

**Figure 2:**
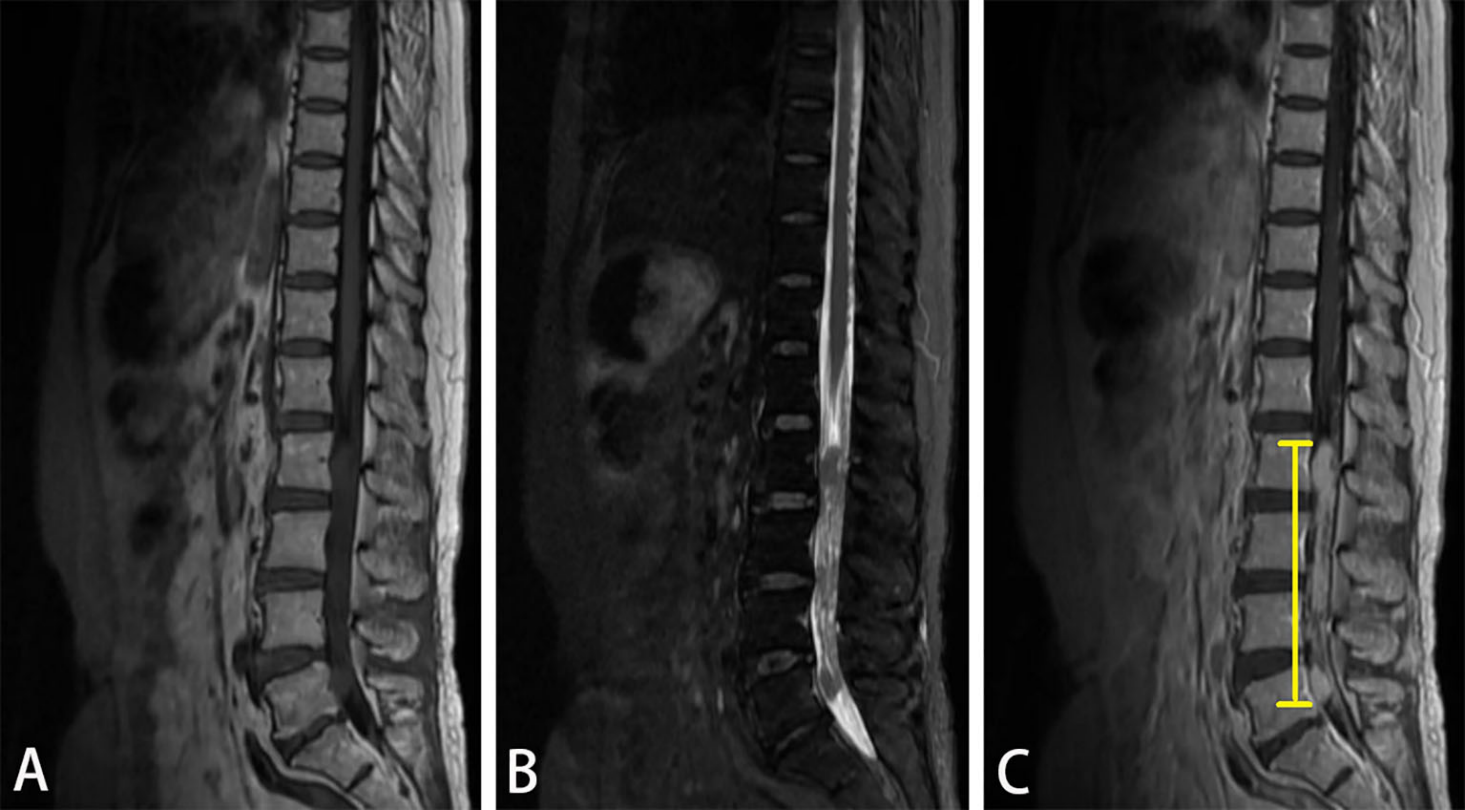
Magnetic resonance imaging (MRI) of the patient’s thoracic and lumbar vertebrae on the admission. Lumbar spine magnetic resonance imaging revealed a space-occupying lesion at the level of the second through fifth lumbar vertebrae. T1-weighted fluid-attenuated inversion recovery MRI **(A)** showed low signal intensity and inhomogeneously low signal intensity on T2-weighted MRI **(B)**. The lesion (indicated by yellow lines) showed a remarkable enhancement on contrast-enhanced T1-weighted MRI **(C)**.

On 1684 d after allo-HSCT, the patient presented pain in the lumbosacral region and numbness in the right lower limb without any obvious cause, and the muscle strength in her limbs was normal. The patient’s pain could not be alleviated after rest. Physical examination on admission revealed spinous process and paravertebral tenderness in the second through fifth lumbar vertebrae. The patient had skin numbness and decreased sensation below the middle and lower third of the right thigh. Therefore, she underwent lumbar MRI on 1712 days after allo-HSCT. The result suggested that there was an abnormal signal intensity in the patient’s spinal canal at the level of the second through fifth lumbar vertebrae, and space-occupying lesions were considered (**Figure 2**). She underwent lumbar intraspinal lesion resection under general anesthesia on 1720 days after allo-HSCT and the lesion was found to be located within the dura.

### Pathological findings

**Figure 3:**
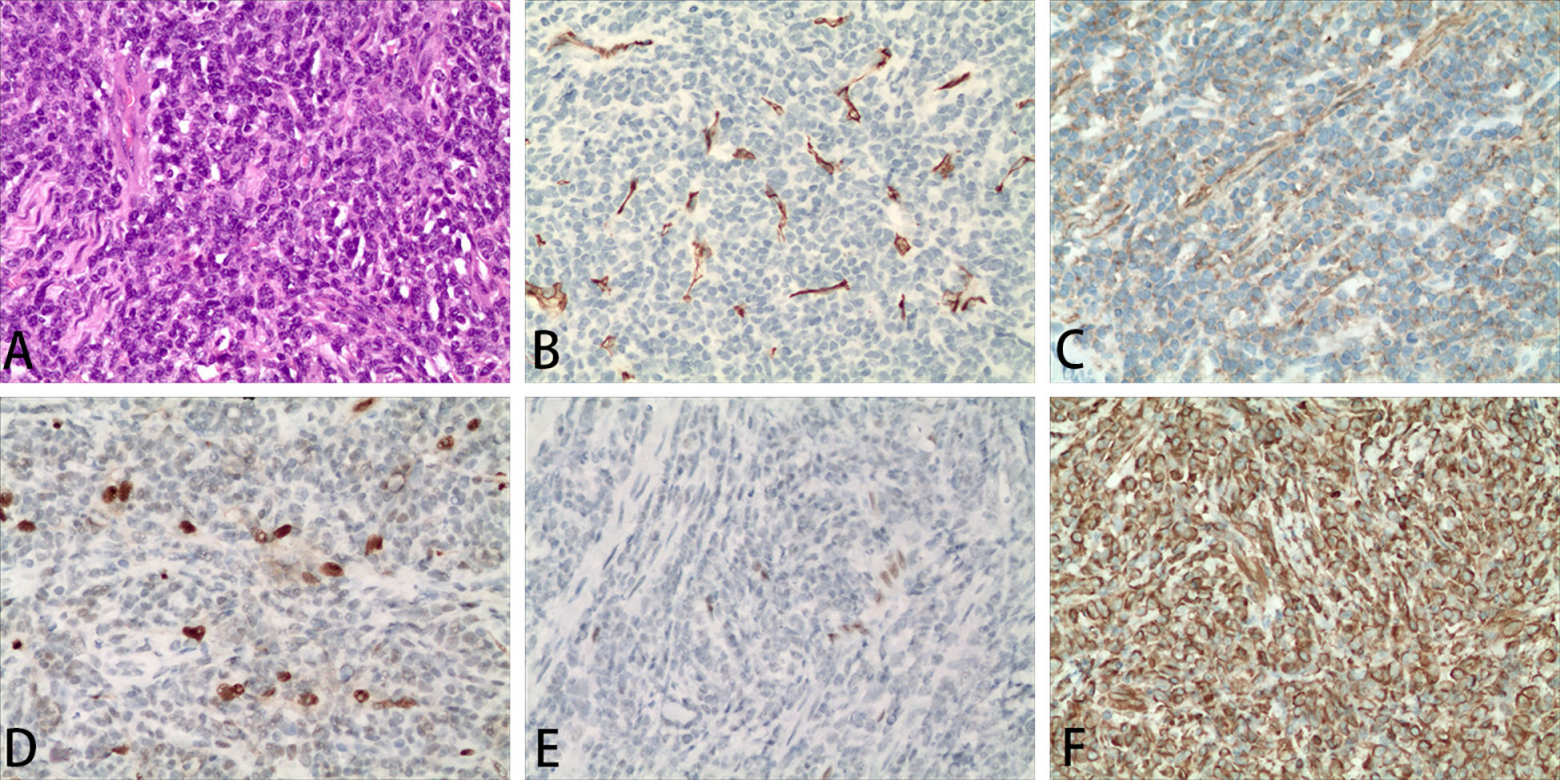
Representative images of the diseased tissue in the lumbar spinal canal of the patient. Hematoxylin and eosin staining of the diseased tissue **(A)**. Representative immunohistochemical staining for CD34 **(B)**, CD56 **(C)**, ERG **(D)**, Fli-1 **(E)**, vimentin **(F)**. Magnification 200×.

The pathological results of the resected tissue in the spinal canal of the patient showed that the tumor cells were densely distributed, with short fusiform cells, round nuclei and inconsistent sizes (**Figure 3A**). The mitotic figures of the tumor cells were >2/10 high-magnification fields and the tumor tissues surrounded the blood vessels. The results of immunohistochemical staining of the patient were as follows (**Figures 3B–F**): tumor cells were positive for vimentin, CD56, Fli-1 (partially weak), ERG (weak), p53 (small amount of weak), Ki67 (30%) while they were negative for CKp, Syn, S-100, SOX-10, CD99, MelanA, Calretinin, alpha-inhibin, GFAP, CD20, CD3, STAT-6, Desmin, SATB-2, LCA, CD34, O1ig-2. The pathological diagnosis of the patient was HPC, WHO grade 3 on 1822 days after allo-HSCT.

### Treatment and follow-up

On 1822 days post-transplant, the patient received radiation therapy to the lesions of the lumbar spinal canal at the level of the second through fifth lumbar vertebrae. Gross tumor volume (GTV) is the location and range of lesions visible on CT. The planning target volume (PTV) was GTV with a 5mm expansion. The PTV prescription dose is 4500cGy/25f. The radiation oncologist reviewed the target volumes and treatment plans. Following radiation therapy, the patient experienced relief from pain and numbness in the lumbosacral region and right lower limb.

## Discussion

It has been reported that the latency period of secondary tumors after allo-HSCT is 3-5 years (6). The 10-year cumulative incidence of secondary tumors after allo-HSCT ranged from 1.0% to 27% (5, 7–11). Several retrospective studies have concluded that the time from transplantation to the occurrence of secondary tumors is 6.7-11.7 years. Secondary tumors are found in patients aged 23.4-61 years after transplantation (5, 10, 11). A large clinical study from Europe enrolled 220,617 HSCT patients over 14 years and found that 1.8% patients developed secondary tumors with a median age at diagnosis of 59.1 years (12).

In our center, 320 patients have received allo-HSCT in the past 10 years and 182 patients have survived for more than 5 years. At present, 1.65% patients after allo-HSCT developed a secondary tumor, with a time range of 4.3 to 11.3 years and a median age of 48.2 years. The time of occurrence of secondary tumors in patients after allo-HSCT in our center was consistent with reports (10, 13). Thyroid cancer is the most common and earliest occurrence after allo-HSCT. Therefore, ultrasounds are recommended to screen for thyroid cancer every year (5, 14).

We report a patient diagnosed with intraspinal anaplastic HPC following allo-HSCT, which is the first reported case. HPC, first described in 1928, is a malignant tumor derived from perivascular cells and usually occurs in bone and soft tissue (15). According to the WHO pathological classification, HPC is WHO grade 2 and anaplastic HPC is WHO grade 3 (16). HPC often occurs in the intracranial space (17, 18) and rarely in the spinal canal (19). Due to the rarity of central nervous system (CNS) HPC and the paucity of randomized clinical trials, there are limited data on this disease and no authoritative guidelines for its management.

Ghose A et al. (18) analyzed 523 patients diagnosed with HPC and found that the average age at diagnosis was about 44 years old. The risk of local recurrence of HPC is high, and HPC may have extroneural metastasis. The most common sites of neurometastasis are lung, bone, liver, intraperitoneal, subcutaneous tissue, breast, pleura, and thyroid. Clinical manifestations of HPC vary according to tumor size and location and are prone to local recurrence after surgical resection (20).

Intraspinal HPC usually presents with sensory and motor abnormalities caused by spinal cord compression or nerve root irritation. Postoperative pathological diagnosis is the gold standard for HPC diagnosis. HPC appears as a typical antler-like vascular pattern, and the tumor cells are obviously deformed, being round, spindle-shaped, or irregular (21). The immunohistochemical manifestations of HPC were positive for CD99, Bcl-2, vimentin, and CD34 (21, 22). In contrast, S100, CD31 and EMA were not expressed (21, 23–25). It has been reported that intraspinal HPC has a high misdiagnosis rate due to lack of specificity in clinical symptoms and imaging examination, and needs to be differentiated from meningioma and schwannoma. According to the location of the tumor, intraspinal HPC can be divided into subdural type, extradural type and extradural type. Surgical resection of intraspinal HPC is the first choice, and vascular embolization is feasible when total resection is difficult.

According to literature, surgical treatment of the lesion site of HPC, especially complete resection (19) and adjuvant radiotherapy (26, 27), has a clear curative effect as first-line treatment (18). Spinal HPC should be removed as a whole to reduce surgical blood loss and possibly increase disease-free survival. Despite surgical removal of these apparently benign lesions, the high recurrence rate requires close follow-up (28). Intraspinal HPC is prone to early recurrence and metastasis (29), and studies have shown that adjuvant irradiation after surgery can reduce the risk of recurrence and metastasis (26, 30). CNS HPC has a poor response to chemotherapy (17), and some studies suggest that HPC outside the CNS may respond well to chemotherapeutic drugs including doxorubicin. In this case, the patient underwent complete surgical resection of the tumor tissue followed by radiotherapy, and the patient’s symptoms of pain and numbness were significantly reduced. This suggests that surgical resection combined with radiotherapy was effective for this patient (31). Notably, Wang K et al. (17) analyzed 1243 patients with HPC and multivariate analysis showed that surgical resection was clinically effective, while the clinical usefulness of adjuvant chemotherapy or radiotherapy is limited. Research shows that antiangiogenic agents, immunotherapy and RNA-targeting technologies are also beneficial for HPC (32), but further clinical studies are needed.

Several studies have confirmed that the prognosis of secondary tumors following allo-HSCT depends mainly on the type of tumor. Studies have reported that the 5-year overall survival rate of secondary pancreatic cancer, lung cancer, hepatobiliary cancer, esophageal cancer, brain cancer and gastric cancer after secondary tumor diagnosis is poor, and the median survival time is between 0.6 and 1 year. The major risk factors for death after secondary tumor diagnosis were age at transplantation, donor type, physical condition, and GVHD (12, 33).

Secondary tumors after allo-HSCT are more complex. Studies have suggested that anti-thymocyte globulin, chronic GVHD, total body irradiation, chemotherapeutic agents such as fludarabine, genetic susceptibility, and viral infection are risk factors for increased secondary tumors after transplantation (5, 34, 35). Skin and oropharyngeal squamous cell carcinoma are associated with chronic GVHD (12). Total body irradiation is mainly associated with an increased risk of non-squamous cell carcinoma (36). Secondary tumors are significantly correlated with radiation dose, and radiation is an important risk factor for breast cancer, thyroid cancer, CNS tumors, bone cancer and melanoma (5, 12). There were 18 cases (18/146) of secondary solid tumors in the total body irradiation based regimen group, much higher than 2 cases (2/280) in the non-irradiation based regimen group (5). Therefore, it has been suggested that in the long-term follow-up of allo-HSCT patients after irradiation, attention should be paid to secondary tumors in young patients (5, 37). There is literature describing potential mechanisms. The use of multiple cytotoxic drugs in the treatment of hematologic diseases, lethal doses of radiotherapy and chemotherapy pretreatment in patients undergoing transplantation, use of immunosuppressants after allo-HSCT, infection, and inflammatory damage caused by GVHD are all risk factors for secondary tumors (38–40).

In conclusion, the use of high-dose cytotoxic drugs, radiotherapy, immunosuppressant and GVHD during allo-HSCT are all related to the development of secondary tumors (12). According to Jacie Standards and international guidelines, screening for secondary tumors after transplantation includes abdominal ultrasound, thyroid ultrasound, and specialized examinations such as otolaryngology, dermatology, gynecology, mammary surgery, and stomatology (8, 35, 41).

## Data Availability

The original contributions presented in the study are included in the article. Further inquiries can be directed to the corresponding author.
